# Chemical trios in rhizosphere ecology: Emerging roles of microbial volatiles, root-derived volatiles, and non-volatile root exudates in plant-soil microbe interactions

**DOI:** 10.5511/plantbiotechnology.25.0210a

**Published:** 2025-09-25

**Authors:** Jun Murata

**Affiliations:** 1 Bioorganic Research Institute, Suntory Foundation for Life Sciences, 8-1-1 Seikadai, Seika, Soraku, Kyoto 619-0284, Japan

**Keywords:** microbial volatile organic compounds (mVOCs), plant-microbe interaction, rhizosphere, root exudates, root volatile organic compounds (rVOCs)

## Abstract

The rhizosphere, a narrow zone of soil directly influenced by plant roots, serves as a highly dynamic interface where biochemical and ecological interactions converge to sustain plant growth. This critical region facilitates intricate chemical exchanges among plants, soil, and microorganisms, thereby shaping nutrient availability, microbial community structures, and plant defense mechanisms. While root exudates that are mostly non-volatile have long been the primal interest as ecologically crucial chemicals in the rhizosphere, recent advancements in analytical methodologies have illuminated the roles of volatile organic compounds produced by soil microorganisms (mVOCs) and plant roots (rVOCs) as intricate mediators that regulate plant physiology and microbial community dynamics. mVOCs exhibit diverse functions, including stimulating root development, enhancing systemic resistance, and suppressing pathogen activity, thereby contributing to plant health. Conversely, rVOCs support soil microorganisms in establishing ecological niches in association with plants. mVOCs and rVOCs, together with root exudates, create feedback loops that drive ecological processes in the rhizosphere and enable plants to adapt to environmental challenges. This review synthesizes current understanding in the composition, molecular mechanisms, ecological relevance, and potential applications of mVOCs and rVOCs, with a particular emphasis on their interplay with non-volatile root exudates. The integration of these insights offers a molecular foundation for advancing sustainable agricultural practices and tackling pressing global challenges, such as ensuring food security and mitigating environmental degradation exacerbated by climate change.

## Introduction

Soil is a biologically rich medium that supports an extraordinary diversity of plants and soil microorganisms. Of the globally estimated species numbers for plants (4.7×10^5^) and microbes (4.4×10^8^), predominantly bacteria, roughly 90% of plant species and 50% of microbial species inhabit soil environments (Anthony et al. 2023). Moreover, approximately 90% of the estimated fungal species (5.6×10^6^) are found in soil (Anthony et al. 2023). Therefore, the majority of plant species inevitably engage with diverse soil microbes at various stages of their lifecycle. Interactions between plants and soil microbes have been extensively studied, with much attention devoted to two areas: the antagonistic dynamics between plants and pathogens, and the mutualistic relationships involving nitrogen-fixing rhizobia and arbuscular mycorrhizal (AM) fungi (Guerrieri and Rasmann 2024; Korenblum et al. 2022; Kumar and Verma 2018; Philippot et al. 2013).

In mutualistic relationships, legumes exemplify a symbiotic system in which plants supply carbon fixed via photosynthesis to rhizobia (Yang et al. 2022). In return, rhizobia fix atmospheric nitrogen and provides it to the plant, creating a mutually beneficial interaction. The molecular basis of this symbiosis is well characterized. It begins with flavonoids and betalains secreted by the plant roots, which are detected by rhizobia. This triggers rhizobia to secrete lipochitooligosaccharides, commonly referred to as Nod factors, which are recognized by LysM receptor-like kinases on the plant. These interactions activate downstream signaling pathways, culminating in the formation of infection threads that allow nitrogen fixation in the root of legumes. Similarly, AM fungi engage in mutualistic associations with a broader range of plant hosts than rhizobia (Bennett and Groten 2022). These fungi form extensive hyphal networks within and across plant tissues, enabling the transfer of carbon from the plant to the fungi. In exchange, AM fungi facilitate the uptake of phosphorus, which is often unavailable in recalcitrant soil forms. This symbiosis is mediated by strigolactones secreted by plant roots, which are detected by the fungi, prompting the formation of infection structures that integrate into the plant’s root tissues.

These interactions predominantly occur within the rhizosphere, a specialized ecological zone that exists between the root surface and the surrounding soil (Prashar et al. 2014). The rhizosphere is divided into three interconnected layers: the *endorhizosphere*, which encompasses the apoplast of the cortex and endodermis, providing direct access to microbes, small molecules, and ions; the *rhizoplane*, which includes the root epidermis; and the *ectorhizosphere*, the outermost layer comprising bulk soil that remains in close proximity to, but does not directly contact with plant roots (York et al. 2016). The rhizosphere is critical for plants, functioning not only as the interface for anchorage, water uptake, and nutrient absorption but also as the primary site of interaction with a diverse array of soil microorganisms. These microbial communities significantly influence plant growth, development, and fitness.

As sessile organisms, plants must rely on sophisticated molecular mechanisms to sense and respond to dynamic changes of microbial communities, mineral ions, water, and various other environmental factors in the rhizosphere to improve their fitness. While many plant-microbe interactions are mediated by non-volatile signaling molecules, including flavonoids, Nod factors, and strigolactones, that are released from roots and soil microbes, recent studies reveal that volatile compounds also play significant roles. For example, various plants emit herbivory-induced plant volatiles (HIPVs) in response to herbivore attacks, enabling neighboring plants and carnivorous arthropods to use these volatiles as signals. This suggests the presence of a complex many-to-many communication network facilitated by HIPVs in the aerial parts of plants (Aartsma et al. 2017; Takabayashi 2022).

On the other hand, recent reports show that plants operate volatile-mediated signaling in the rhizosphere as well; various microbial and root- volatile organic compounds (mVOCs and rVOCs) have been found to exhibit diverse biological activities that profoundly influence plant growth and development. Due to the substantially greater biodiversity in the rhizosphere compared to the phyllosphere—the surface of aerial plant parts—plants are likely to engage in significantly more intricate many-to-many communication within the rhizosphere (Anthony et al. 2023; Dong et al. 2019). This review explores the biological and ecological relevance of mVOCs and rVOCs with particular emphasis on their relationship with non-volatile root exudates in shaping interactions between plants and soil microbes.

## mVOCs as part of the global carbon cycle

The global carbon cycle, as assessed by the U.S. Department of Energy, involves the absorption of approximately 120 Gt of carbon annually by plants through photosynthesis, predominantly in the form of atmospheric carbon dioxide (https://www.energy.gov/science/doe-explainsthe-carbon-cycle). Of this absorbed carbon, 60 Gt is fixed into plant tissues, while the remaining 60 Gt is released back into the atmosphere. This release occurs in several forms, including carbon dioxide via respiration and volatile organic compounds (VOCs), which are typically defined as organic molecules with low molecular weight, generally ranging from 50 to 300 Da, such as isoprene, emitted for example from conifer leaves under heat stress, and *cis*-3-hexenol, a “green leaf volatiles” emitted by leaves damaged by herbivorous insects (Brosset and Blande 2022; Takabayashi 2022; Vincenti et al. 2019).

Notably, the carbon cycle in plants extends beyond the above-ground components to include processes occurring in root systems and the surrounding soil. Majority of the organic carbon fixed in plant tissues is not permanently sequestered. Approximately 5–21% of the carbon that is fixed during photosynthesis is released into the rhizosphere as rVOCs and other non-volatile organic molecules, the latter of which are generally called as “root exudates” (Simon and Haichar 2019). Moreover, plant residues decompose in the soil, where microbial activity further metabolizes the organic material. These microbial processes release carbon back into the atmosphere as mVOCs. Thus, beyond exposure to HIPVs and rVOCs from plant sources, plants also encounter mVOCs. This suggests that plants have evolved to adapt to environments in which they coexist with diverse VOCs, including mVOCs derived from microbial metabolism. As global temperatures rise due to climate change, soil temperatures are projected to increase, potentially altering the structure and metabolic profiles of soil microbial communities (Metcalfe 2017). Such shifts are likely to influence mVOC production, with cascading effects on plant growth and ecosystem dynamics.

## Soil bacteria and fungi as producers of mVOCs

mVOCs are chemically diverse volatile molecules consisting of various classes of compounds such as alcohols, aldehydes, organic acids, esters, ketones, and terpenoids (Russo et al. 2022). This structural diversity is mirrored in their functional roles, which range from promoting plant growth and enhancing disease resistance to inhibiting competing microorganisms. The result of technological advancements, including the increased sensitivity and accuracy of gas chromatography-mass spectrometry (GC-MS) and liquid chromatography-mass spectrometry (LC-MS) as well as development of various “-omics” approaches and databases, have enabled the characterization of an array of mVOCs that would have been otherwise undetectable nor identifiable.

Indeed, the recently developed “mVOC 4.0” database (https://bioinformatics.charite.de/mvoc/ (Accessed Jan 9, 2025)), constructed through extensive text mining of scientific literature, has significantly improved our ability to annotate these compounds (Kemmler et al. 2024; Lemfack et al. 2018). The database allows users to search for mVOCs based on their chemical properties, microbial origin, and effects on plant species, while also enabling the identification of bacterial or fungal producers from given mVOC chemical profiles. Conversely, mVOC 4.0 also helps users to identify specific bacterial or fungal species by analyzing the chemical profile of mVOC samples.

The physiological functions of mVOCs are highly diverse, with many studies documenting their ability to promote plant growth, enhance stress tolerance, and suppress pathogens (Russo et al. 2022; Veselova et al. 2019). Soil bacteria known as plant growth-promoting rhizobacteria (PGPR) have emerged as valuable sources of mVOCs that are agriculturally valuable particularly in promoting the growth of monocots such as grasses, dicots like solanaceous crops, and woody perennials such as poplar, many of which do not form symbiotic relationships with rhizobia or AM fungi (Russo et al. 2022).

Unlike synthetic pesticides or fertilizers, PGPRs are relatively easy to propagate, and appeal to organic agricultural systems due to their compatibility with natural plant-microbe interactions (Basu et al. 2021). These attributes make them ideal candidates for developing sustainable agricultural input. However, while many studies have documented the general effects of PGPRs, research into the identification of specific mVOCs responsible for these effects remains limited. Most reports describe chemical profiling of mVOCs released from PGPRs with GC-MS, without purification or identification of the individual volatiles responsible for the observed growth promotion.

In a seminal study, Ryu et al. demonstrated that mVOCs such as 2,3-butanediol, released by *Bacillus subtilis* (GB03) and *Bacillus amyloliquefaciens* (IN937a), significantly enhanced the growth of *Arabidopsis thaliana*. The study also showed that bacterial strains lacking the ability to produce 2,3-butanediol failed to promote growth, highlighting the compound’s essential role (Ryu et al. 2003). Similarly, dimethyl disulfide produced by *Bacillus* spp. B55 has been shown to promote the growth of *Nicotiana* plants, further supporting the importance of bacterial volatiles in plant development (Meldau et al. 2013). Although *Bacillus* spp. dominates the literature on PGPRs, other bacterial genera, including *Pseudomonas*, have also shown potential as producers of plant growth-promoting mVOCs (Russo et al. 2022). However, the diversity of soil bacteria, estimated to exceed plant species by a factor of 1,000 (Lee et al. 2015), suggests that a vast reservoir of unexplored mVOCs and their microbial producers remains to be studied. Systematic exploration of bacterial species, combined with targeted investigation of their associated mVOCs, holds a significant promise for identifying novel compounds and elucidating mechanisms that enhance plant signaling.

Fungi, although less extensively studied than bacteria in the context of mVOCs production, has also been identified as significant contributors to plant growth promotion. Notable examples include species from the *Trichoderma* genus, as well as certain *Streptomyces* species (Russo et al. 2022). These fungi release a range of mVOCs, including 4-heptanone, isobutyl alcohol, undecanal, and isocaryophyllene, which have been implicated in enhancing plant development and stress tolerance (Liao et al. 2013; Nieto-Jacobo et al. 2017). However, fungal diversity is estimated to be one-tenth of bacterial diversity, with approximately 5.6×10^6^ species globally (Anthony et al. 2023). This lower diversity suggests that fungi are a narrower, but potentially high-impact, target for discovering new mVOCs with agricultural applications.

Despite the substantial roles of PGPRs and fungi in promoting plant growth, there are ecological challenges in translating laboratory findings to field conditions. For instance, in many cases, introduced microbial strains fail to establish themselves in the soil or are outcompeted by native microbial communities, limiting their efficacy (Garbeva et al. 2014). Ensuring the survival and persistence of selected strains in field conditions is therefore a critical step in the development of effective microbial inoculants. Controlled environments, such as plant factories, offer a promising alternative for harnessing the benefits of PGPRs and their mVOCs under well-regulated conditions (Conant et al. 2017).

While most studies on mVOCs have focused on their positive effects, it is important to note that not all mVOCs promote plant growth. Some mVOCs, such as ammonia released by *Serratia odorifera*, have been shown to inhibit root elongation in *A. thaliana*. Similarly, ketones including 2-nonanone, 2-heptanone, and 2-undecanone, emitted by certain bacterial strains like *Pseudomonas chlororaphis*, negatively impact plant development (Weise et al. 2013). Furthermore, pathogenic fungi produce mVOCs that can disrupt plant metabolism, inhibit growth, and compromise fitness (Thankappan et al. 2022). Compared to growth-promoting mVOCs, these inhibitory compounds are less extensively studied and remain poorly understood. Recent research has offered deeper insights into the mechanisms of how selected mVOCs negatively affect plant growth.

For example, co-culturing *A. thaliana* with *Bacillus atrophaeus* (formerly *B. subtilis*) ATCC9372 resulted in significant growth inhibition. Using bio-organic chemistry approaches, isovaleric acid was identified as the active inhibitory compound (Murata et al. 2022). Isovaleric acid inhibited primary root elongation. Structurally related compounds such as isobutyric acid exhibited similar effects. Interestingly, naturally occurring (*S*)-2-methylbutyric acid exhibited stronger inhibitory activity than its synthetic (*R*)-enantiomer. This suggests that plants have evolved specific recognition mechanisms that distinguish differences in chirarity and functional groups of mVOCs. The ecological and biological significance of inhibitory mVOCs, such as isovaleric acid, is still an open question. For *Bacillus atrophaeus*, the release of such compounds may confer competitive advantages in microbial ecosystems.

Growing evidence indicates that mVOCs influence plant physiology either at the post-transcriptional or post-translational level, highlighting the vast diversity in how plants recognize these compounds (Ameztoy et al. 2019; García-Gómez et al. 2019). Understanding the molecular basis of plant recognition of mVOCs could provide (i) insights into developing crop varieties with enhanced resilience or increased sensitivity to inhibitory or growth-promoting mVOCs, thereby improving agricultural productivity and sustainability, and (ii) clues to uncover the biological and ecological reasons behind the evolution of isovaleric acid and other “multivalent metabolites (i.e., metabolites with multiple functional roles)” as species-specific functional molecules found across all three kingdoms of life (Ono and Murata 2023).

In summary, while mVOCs from both bacteria and fungi offer immense potential for enhancing plant growth and resilience, their dual roles as growth promoters and inhibitors highlight the complexity of plant-microbe interactions. It is of particular interest to determine whether rhizobia and arbuscular mycorrhizal (AM) fungi utilize mVOCs as signaling molecules that act earlier in the interaction process than non-volatile compounds such as Nod-factors and strigolactones. Future research should aim to uncover the full diversity of mVOC-producing microbes and their functional roles and use this knowledge to design innovative agricultural solutions that balance productivity and environmental sustainability.

## Plant-derived root volatile organic compounds (rVOCs) are also biologically relevant in rhizosphere communication

Soil microorganisms are not the only contributors to the rhizosphere “volatilome”. Recent studies provide critical insights into the vital roles played by a blend of VOCs that are derived from plant roots as well. Such rVOCs, which are released into the rhizosphere together with non-volatile root exudates, have been shown to coordinate plants’ responses to various environmental cues, including the ones either from beneficial or pathogenic soil microorganisms, and help microbial communities adapt to the rhizosphere (Pascale et al. 2020). For example, rVOCs can attract beneficial microorganisms like PGPR, enhancing plant growth, nutrient acquisition, and disease resistance (Russo et al. 2022). On the other hand, mVOCs emitted by pathogenic fungi can inhibit plant growth, damage plant tissues, and compromise plant defenses (Thankappan et al. 2022). These contrasting roles of rVOCs and mVOCs are central to VOC-mediated plant immune responses and overall plant health, making them a critical aspect of plant-microbe signaling in the rhizosphere.

Recently, volatile methyl jasmonate (MeJA), emitted by roots of *Arabidopsis* plants, was shown to enhance soil microbiome biofilms and found that MeJA is a key bioactive signal that promotes biofilm formation in soil microorganisms (Kulkarni et al. 2024). These biofilms, compared to planktonic microbial populations, provide enhanced ecological benefits, including nutrient cycling and plant growth promotion. Importantly, this MeJA-induced biofilm formation is evolutionarily conserved across diverse plant taxa, suggesting its universal role in rhizosphere interactions. The study underscores MeJA’s potential in sustainable agriculture to manipulate soil microbiomes for improved plant performance.

Conversely, rVOCs also mediate indirect interactions between plants and fungi through bacterial intermediaries (Duc et al. 2022). For example, *Carex arenaria* roots when infected with fungal pathogen *Fusarium culmorum* change the profile of rVOCs so that *Paenibacillus* sp. and *Collimonas* sp. of bacteria with antifungal activity were attracted towards *Carex* roots (Schulz-Bohm et al. 2018). Similarly, roots of tomato plants infected by pathogenic *F. oxysporum* release naphthalene, *p*-cymene, and 3-carene that have antifungal activities and also emitted yet to be identified rVOCs that attract beneficial *Bacillus* spp. (Gulati et al. 2020). These studies underline the ecological and agricultural significance of rVOCs and highlight dual roles of rVOCs that are likely widespread across plant kingdom in enhancing beneficial plant-microbe interactions and deterring pathogens.

## Expanded roles of root exudates beyond allelochemicals between plants

Root exudates are composed of non-volatile biochemically diverse compounds, including carbohydrates, organic acids, and amino acids, and proteins that are not readily transformed into the gaseous phase under ambient temperature and pressure (Afridi et al. 2024). Root exudates play multiple roles in the ecosystem. On a global scale, they serve as a vital carbon source for soil microbes, contributing to the carbon cycle. On the other hand, their biological significance has been associated with allelopathy, where the exudates of one plant species inhibit the growth of neighboring species. This process is thought to enhance the plant’s fitness by reducing competition in its environment (Bertin et al. 2003; Hierro and Callaway 2021; Scavo et al. 2019; Wang et al. 2021; Weir et al. 2004). A diverse array of chemicals has been identified as allelochemicals from various plant species typically in a lineage-specific manner.

Moreover, recent report revealed that wheat roots trigger the secretion of an allelochemical 2,4-dihydroxy-7-methoxy-1,4-benzoxazin-3-one (DIMBOA) when exposed to (−)-loliolide and jasmonic acid that are secreted from neighboring plants of various non-wheat species, showing an example of how plants sense the existence of nearby conspecific plants (Kong et al. 2018; Wang et al. 2021). These findings imply that (−)-loliolide and jasmonic acid are recognized by wheat plants as signals for plant-plant communication.

On the other hand, increasing evidence shows that root exudates not only function as allelochemicals against other plants but also play important roles as chemical signals that shape microbial community composition and functionality. Root exudates are crucial for plant development and have various biological activities in the rhizosphere including mineral acquisition, antimicrobial activities against pathogens, and recruitment of competitors against pathogens (Hong et al. 2022). Root exudates facilitate nutrient acquisition, particularly nitrogen and phosphorus, through symbiotic interactions with rhizobia and mycorrhizal fungi, respectively (Chai and Schachtman 2022; Guerrieri and Rasmann 2024; Korenblum et al. 2022; Kumar and Verma 2018). Importantly, root exudates often include phytosiderophores, which are specialized compounds capable of binding and mobilizing minerals in the soil.

For instance, plants in the Poaceae family release mugineic acids, a group of compounds that function as iron (Fe) chelators (Curie and Briat 2003; Kobayashi et al. 2019). Fe, although the fourth most abundant element in soil, mostly exists as insoluble Fe(III), thus necessitating the secretion of Fe chelators in securing the productivity of plants especially in calcareous soil where the solubility of Fe decreases even more. Furthermore, root exudates influence soil physicochemical properties and regulate microbial community structure, impacting plant growth and development (Molefe et al. 2023). Through these interactions, plants selectively recruit beneficial microbes while suppressing pathogenic organisms. This selective recruitment, often referred to as “cry-for-help signaling”, allows plants to modulate their exudation chemistry in response to biotic and abiotic stressors. By doing so, plants establish a balanced microbial ecosystem that supports growth, enhances immunity, and ensures survival under challenging environmental conditions (Pascale et al. 2020; Rolfe et al. 2019).

Remarkably, the biological importance of root exudates is not only limited to inter-species interactions but is also evidenced in establishing intra-species, female plant-specific communications in poplar, a dioecious plant (Xia et al. 2023). Female roots of dioecious *Populus cathayana* release a greater amount and more diverse phenolic compounds, including (−)-epigallocatechin, 4-methoxycinnamic acid, hesperetin and other flavonoids, into the soil, resulting in growth inhibition of female neighbors (Xia et al. 2023). It is notable that, compared with female monoculture set up, female plants when grown with males reduced the level of total phenolic compounds in the soil under such experimental conditions. This biochemical change in root exudate profiles lead to an ecological shift from allelopathic inhibition to chemical facilitation in the relationship between plants.

More surprisingly, the change in the root exudate profiles was associated with the change in the soil microbiota as well as the directions of root elongation, both seemingly being favorable in increasing the fitness of female plants (Xia et al. 2023). Moreover, *Populus euphratica* roots were shown to release various organic acids, including oxalic acid and glutaric acid, in a sex- and season-dependent manner (Liu et al. 2024). These organic acids in root exudates likely contribute to solubilizing otherwise recalcitrant phosphorus in soil. As in the case of *P. cathayana*, differences in the profiles of root exudates coincide with the differences in microbiota, specifically the abundance and the richness of AM fungi and phosphate-solubilizing bacteria in surrounding soil (Liu et al. 2024). Therefore, the data implies that female and male plants of *P. euphratica*, as a dioecious plant species, might have established an ecological strategy to share limited source of phosphorus using sex-dependent differences in the season of release as well as the profiles of root exudates, likely preferring female and male plants to co-inhabit in a proximity and maintain their dioecious system.

Additionally, research on intercropping shows that the benefits of this practice on plant growth and nutrient uptake are driven largely by interactions between rhizosphere metabolites and microbial communities (Jiang et al. 2024). Indeed, the exogenous application of key metabolites enriched by intercropping significantly promoted maize biomass in natural soil, but not in sterilized soil, further underlining the potential of chemical-selected microbiomes to modulate plant health and illustrate that both positive and negative plant-soil feedback observed in nature are, at least to some extent, microbiota-dependent (Jiang et al. 2024).

## Dynamic patterns in the distribution of mVOCs, rVOCs, and root exudates

mVOCs, rVOCs, and root exudates are critical players in the intricate biochemical and ecological dynamics of the rhizosphere. These compounds facilitate a wide range of interactions between plants and soil microorganisms, influencing nutrient acquisition, microbial community composition, and stress adaptation, as shown in this review. Importantly, the biosynthesis and release of rVOCs and root exudates are not passive processes. Rather, they are tightly regulated by developmental stage, physiological state, and external environmental cues such as nutrient availability, soil pH, and light cycles of plants (Canarini et al. 2019; Pascale et al. 2020). This spatio-temporal regulation of rVOCs and root exudates secretion underscores the plant’s ability to dynamically adjust its chemical environment to improve fitness and survival.

### Diurnal biosynthesis and release of root exudates and rVOCs

Biosynthesis and secretion of mugineic acids in Poaceae plants provide an excellent example of how plants actively modulate root exudation in response to environmental demands. Mugineic acids, a group of phytosiderophores, are specialized metabolites that are synthesized through the conjugation of three methionine molecules. This process is catalyzed by enzymes in the methionine cycle, followed by the production of nicotianamine, which is then converted to mugineic acids. These compounds are secreted into the rhizosphere via ATP-binding cassette (ABC) transporters (Kobayashi and Nishizawa 2012; Nozoye et al. 2004).

Notably, biosynthesis and secretion of mugineic acids are regulated by the plant’s internal circadian clock. Research shows that mugineic acid secretion peaks at the dawn, coinciding with the plant’s enhanced capacity to absorb Fe-chelates (Nozoye et al. 2004). This circadian periodicity aligns with environmental water potential dynamics driven by transpiration and photosynthesis in the aerial parts of the plant. During daylight hours, water loss through transpiration creates an upward water stream in the rhizosphere, helping the transport of solubilized Fe(III)-mugineic acid complexes from the rhizosphere to the root surface for uptake. The synchronization of metabolic activity, environmental rhythms, and nutrient transport highlights the intricate regulation of root exudates in response to both temporal and spatial environmental changes (Nozoye et al. 2004; Kobayashi et al. 2019).

This dynamic regulation suggests that soil microbes near the roots of Poaceae plants experience the diurnal secretion of mugineic acids. While soil bacteria typically produce their own siderophores to chelate Fe(III), some bacteria utilize siderophores produced by other microorganisms instead of synthesizing their own (D’Onofrio et al. 2010; Singh et al. 2022). This raises ecologically significant questions: Are Poaceae plants and soil bacteria primarily competing or collaborating over iron resources, particularly during the early hours of the day? Furthermore, could there be soil microbes preferentially adapted to the periodic “once-a-day shower” of plant-derived siderophores, rather than relying on bacterial siderophores? Addressing these questions could deepen our understanding of plant-microbe interactions and resource dynamics in the rhizosphere.

More recently, soybean roots were shown to regulate the diurnal secretion of specialized metabolites. Isoflavones such as daidzein and genistein, along with their malonyl glycosides, peak in the morning, whereas soyasaponins are secreted primarily in the afternoon (Matsuda et al. 2020). These findings suggest that plants adjust the composition of root exudates based on the biological roles of these metabolites, aligning their secretion with specific functional demands. Furthermore, metabolomic analysis of root exudates from *A. thaliana*, *Brachypodium distachyon*, and *Medicago truncatula* identified that, apart from the species-specific metabolites, there are chemicals that are commonly found across these three distinct plant species, showing that selected sets of metabolites might play general roles in establishing favorable rhizosphere environment (McLaughlin et al. 2023). Notably, the core metabolome of root exudates, consisting of over 30 metabolites, includes various amino acids, carbohydrates, nucleotides, and organic acids, and many of them were under the control of circadian periodicity.

Release of rVOCs is also under the control of diurnal rhythm. The amount of total rVOCs showed approximately two-fold increase during daytime compared to nighttime in rapeseed and tomato plants, leaving the possibility that the rise in temperature during the day could partly contribute to the release of rVOCs from soil (Voyard et al. 2024). Therefore, there is no surprise if rVOCs shape the growth and viability of soil microbes diurnally as in the case of mugineic acids and other root exudates.

### Developmental regulation of root exudates secretion

The release of root exudates is developmentally regulated (Chaparro et al. 2013; Loo et al. 2024; Zhalnina et al. 2018). This exudation process is far from static, being shaped by intrinsic root traits, the stratification of soil horizons, and external factors such as climatic conditions (Maitra et al. 2024). These influences collectively create a dynamic interplay between plant physiology and the surrounding soil environment, making root exudation a biological process that is tightly linked to changes in the soil environmental factors. Over the course of plant development, the composition of root exudates undergoes significant changes. For instance, sugars typically dominate the early exudation profile but gradually decline as plants mature, while amino acids and phenolic compounds increase in abundance (Chaparro et al. 2013). These temporal shifts in exudate composition reflect the plant’s changing metabolic needs and its interaction with soil microbial communities.

In the rhizosphere, root exudates build a metabolic microenvironment that is unique to each plant species, selectively enriching microbial populations based on their substrate preferences and metabolic capabilities. For example, certain sugars may promote the proliferation of beneficial rhizobacteria, while phenolics could act as antimicrobial agents, suppressing pathogens (Zhalnina et al. 2018). Plants actively regulate the release of various sugars, amino acids, and organic acids through mechanisms such as source/sink dynamics and the activity of specific efflux carriers (Canarini et al. 2019). Recent report showed that microbiota, expression profiles of a family of SWEET sugar transporters favoring different substrates to secrete to the soil, and the profile of root exudates, sugars and organic acids in particular, were spatially correlated along the longitudinal axis of the root (Loo et al. 2024). Notably, metabolic profiles and endospheric root microbiota are developmentally discrete across the longitudinal axis of the root and such profiles were rearranged in *sweet* mutant plants. These results show that profiles of root metabolites and microbial colonization are tightly linked and mutually dependent.

Why plant roots precisely secrete different profiles of root exudates along their elongation axis and root microbiota needs to be spatially organized are currently open questions. Moreover, root exudation is deeply connected to broader ecological and agricultural outcomes. As plants adapt their exudation profiles to cope with environmental changes—such as drought, salinity, or nutrient deficiencies—they can influence not only their immediate microbial partners but also the overall ecosystem function. Understanding the dynamics of root exudation is therefore critical for developing strategies to enhance plant adaptation to climate change, conserve biodiversity, and promote sustainable agricultural practices (Canarini et al. 2019; Maitra et al. 2024). For instance, using knowledge of how root exudates facilitate plants to adapt to their soil environment could lead to the design of crops that are more efficient in nutrient use or better equipped to recruit beneficial microbes, reducing the need for chemical fertilizers and pesticides.

### “Remote” vs. “local” signals in the soil; diffusibility of VOCs and root exudates

**Figure 1:**
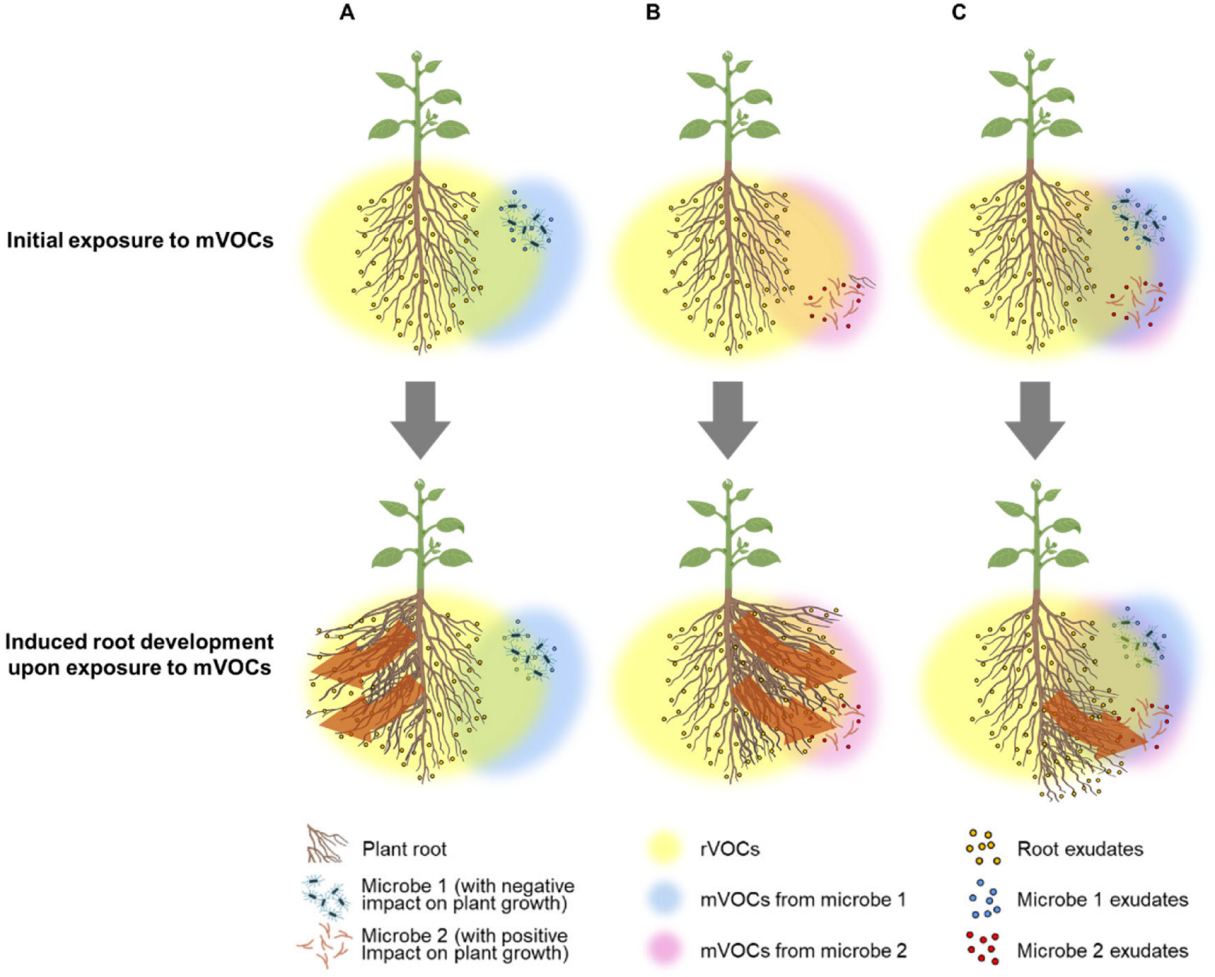
Figure 1. Conceptual illustrations of VOC-mediated establishment of plant-microbe interactions. (A) Bipartite interaction between plants and microbe 1 with a negative impact on plant growth. In this interaction, rVOCs released by plant roots reach the microbe, while mVOCs produced by the microbe reach plant roots at distances where root exudates and microbial exudates remain spatially distinct and do not directly interact. Exposure to mVOCs induces root development in a direction away from the microbe. (B) Bipartite interaction between plants and microbe 2 with a positive impact on plant growth. Here, rVOCs released by plant roots reach the microbe, and mVOCs emitted by the microbe reach plant roots at distances where root exudates and the microbe exudates do not come into direct contact. Exposure to mVOCs induces root development in a direction towards the microbe and subsequently establish more intimate association between plants and the microbe. (C) Tripartite interaction involving plants, microbe 1, and microbe 2. In this scenario, rVOCs from plant roots reach both microbes 1 and 2, while mVOCs from both microbes reach plant roots at distances where exudates from plants and the two microbial species remain isolated without direct interaction among the involved organisms. Physiological responses to mVOCs and rVOCs are determined by whether these volatile compounds exert positive or negative effects on the interacting organisms. Therefore, the root system is developed in a spatially more localized manner. The figure was created based on the resources available at BioRender.com (https://app.biorender.com/illustrations/677c8d6788dcded8884812cd)

The dynamic interplay between mVOCs, rVOCs, and root exudates further enriches rhizosphere interactions. Root exudates primarily influence processes close to the root, such as nutrient mobilization and attracting microbes. Indeed, an isoflavone daidzein which is secreted from roots of soy plants and recruits rhizobium for developing root nodule symbiosis, is estimated to diffuse no farther than 2 mm from root surface (Okutani et al. 2020). In contrast, mVOCs and rVOCs, because of their volatile nature, spread over much longer distances than non-volatile compounds in the soil. In an experimental set up where various microorganisms were introduced into soil at a distance of 4.6 cm from the rVOCs of *Carex arenaria* (sand sedge), under nutrient poor conditions, *Burkholderia*, *Dyella*, and *Pseudomonas* spp. of bacteria were significantly attracted to plant roots, whereas under nutrient rich conditions, *Paenibacillus* spp. exhibited similar root-directed movement (Schulz-Bohm et al. 2018). Additionally, when plants were infected with the fungal pathogen *Fusarium* spp., a mycophagous *Collimonas* spp. of bacteria was found to be more strongly attracted to the roots. Furthermore, the analysis of the diffusion of the authentic standards for mVOCs from *F. culmorum* and rVOCs from *Carex* showed that selected mVOCs and rVOCs spread as long as 12 cm (Schulz-Bohm et al. 2018). From these findings, it has been suggested that plants may employ a strategy to recruit beneficial soil microorganisms by releasing rVOCs, particularly under pathogen attack, to suppress the growth of harmful fungi like *Fusarium*. However, considering that the reported elongation rate of plant roots (8.6 to 74 mm per day) is more than 10 times faster than that of *Rhizoctonia* hyphae (0.2 to 5 mm per day), it is more plausible that plant roots grow toward favorable directions in the soil by detecting mVOCs, microbial exudates, and the spatial distribution of minerals, ultimately encountering microbial colonies, rather than actively recruiting beneficial microbes while deterring unfavorable ones (Watt et al. 2006) (Figure 1). The broader spatial reach enables mVOCs and rVOCs to function as “remote” chemical signals over the distances that are much longer than those of “local” root exudates, influencing microbial behavior and plant responses in the surrounding soil matrix (Schulz-Bohm et al. 2017).

### Mutual regulation of the biosynthesis and release of mVOCs and root exudates

Evidence suggests that mVOCs and root exudates may mutually influence their biosynthetic pathways, creating feedback loops that integrate plant and microbial signaling networks. For instance, certain mVOCs emitted by beneficial microbes can stimulate the production of specific root exudates, enhancing nutrient availability or pathogen suppression (Russo et al. 2022; Stringlis et al. 2018). Conversely, root exudates can alter microbial metabolic activity (Loo et al. 2024), which therefore has a massive impact on modulating the emission profiles of mVOCs and their ecological functions. Furthermore, profiles of rVOCs vary significantly between healthy and diseased plants, with distinct blends attracting biocontrol agents and PGPR. These recruited microbes, in turn, release their own mVOCs, reinforcing plant defenses and establishing a protective rhizosphere microbiome (Almeida et al. 2022; Naik et al. 2024). Understanding the regulatory mechanisms governing the interplay between root exudates and mVOCs holds significant promise for advancing agricultural practices. By manipulating the biosynthetic pathways of these compounds, it may be possible to engineer crops with enhanced nutrient acquisition, stress tolerance, and disease resistance. Such approaches could reduce the reliance on synthetic fertilizers and pesticides, promoting more sustainable farming systems.

## Challenges for the practical application of mVOCs, rVOCs, and root exudates as agricultural tools

The functional versatility of mVOCs, rVOCs, and root exudates offers substantial potential for advancing sustainable agricultural practices. As concerns over the environmental impacts of synthetic agrochemicals intensify, more eco-friendly approaches utilizing natural mVOCs and root exudates are gaining traction. For example, mVOCs can be formulated into bioinoculants that promote plant growth, suppress pathogens, and support sustainable farming systems. These applications present a viable path to reducing agriculture’s environmental footprint while addressing global food security challenges (Thankappan et al. 2022). Despite considerable progress, several challenges remain in elucidating the molecular mechanisms and ecological roles of mVOCs and root exudates.

One key challenge lies in their immense chemical diversity. While approximately 1,000 mVOCs have been found so far, this represents only a small fraction of their true diversity. Advanced analytical techniques, including metabolomics and omics-based approaches, are essential for uncovering the specific roles of these compounds in plant-microbe interactions (Kemmler et al. 2024; Piechulla and Degenhardt 2014). Another challenge is the context-dependent nature of mVOCs activity, as their effects vary widely with environmental conditions such as nutrient availability, temperature, and soil composition, as well as their concentration and application mode. Addressing these complexities requires interdisciplinary research that integrates molecular biology, microbial ecology, and agronomy to develop a comprehensive understanding of mVOCs functions in diverse settings as previously suggested (Piechulla and Degenhardt 2014).

Climate change further complicates this field, as shifts in temperature, precipitation, and atmospheric composition are expected to alter microbial metabolism and mVOCs production. These changes could profoundly affect plant-microbe interactions, with cascading implications for crop resilience and ecosystem stability. For example, ozone exposure has been shown to influence microbial activity and plant growth, underscoring the need to consider atmospheric factors in rhizosphere research (Agathokleous et al. 2020; Kulkarni et al. 2024). Understanding how climate change influences mVOCs dynamics is crucial for developing adaptive agricultural strategies that mitigate its impact while using the benefits of mVOCs.

## Summary

This review highlights the biological and ecological importance of mVOCs, rVOCs, and root exudates in plant growth and development. Elucidation of the molecular mechanism of how plants sense mVOCs and integrate mVOCs signals into their physiological processes is a primal research area for future investigation (Figure 1). Additionally, the biological significance of mVOCs production for microorganisms themselves stays a topic of considerable interest. These compounds may serve diverse ecological functions, from microbial competition to the establishment of beneficial/ecological plant-microbe symbioses.

Beyond the effects of mVOCs on plant growth, recent studies have underscored the importance of broader environmental factors in mVOCs dynamics. These findings highlight the intricate interplay between plants, microbes, and their environment, mediated by mVOCs and root exudates. As evidence mounts for the critical roles of mVOCs and root exudates in plant-soil microbe interactions, the identification of specific mVOCs and root exudates, and their target molecules has become more crucial. Compared to mVOCs, much remains to be understood about the bioactivity and chemical structures of rVOCs, highlighting the need for further investigation into their physiological roles and molecular characteristics. In addition, there exists substantial challenges in *in situ*, non-destructive sampling and quantification of each chemicals of mVOCs, rVOCs, and root exudates from soil (Insam and Seewald 2010; Vives-Peris et al. 2020; Yin et al. 2020). Furthermore, not much has been elucidated about the dynamics and specificity of soil as potential sources and sinks of mVOCs and rVOCs (Jiao et al. 2023). Addressing these issues in the sampling process and unraveling the broader ecological functions of these compounds are key steps toward fully understanding the ecological significance of mVOCs, rVOCs, and root exudates, paving the way for sustainable agricultural practices that are resilient to global climate change

## Abbreviations

AM: arbuscular mycorrhiza
HIPVs: herbivory-induced plant volatiles
MeJA: methyl jasmonate
mVOCs: microbial volatile organic compounds
PGPR: plant growth-promoting rhizobacteria
rVOCs: root volatile organic compounds
VOCs: volatile organic compounds
